# Anti-CEA-functionalized superparamagnetic iron oxide nanoparticles for examining colorectal tumors *in vivo*

**DOI:** 10.1186/1556-276X-8-413

**Published:** 2013-10-08

**Authors:** Kai-Wen Huang, Jen-Jie Chieh, In-Tsang Lin, Herng-Er Horng, Hong-Chang Yang, Chin-Yih Hong

**Affiliations:** 1 Department of Surgery and Hepatitis Research Center, National Taiwan University Hospital Taipei, 100, Taiwan; 2 Graduate Institute of Clinical Medicine, National Taiwan University, Taipei 100, Taiwan; 3 Institute of Electro-optical Science and Technology, National Taiwan Normal University, Taipei 116, Taiwan; 4 Center for Molecular Imaging and Translational Medicine, Xiamen University, Xiamen 361, China; 5 Graduate Institute of Electronics Engineering, National Taiwan University, Taipei 106, Taiwan; 6 Department of Electro-Optical Engineering, Kun Shan University, Tainan 710, Taiwan; 7 Graduate Institute of Biomedical Engineering, National Chung Hsing University, Taichung 402, Taiwan

**Keywords:** Carcinoembryonic antigen, Magnetic nanoparticles, Scanning superconducting-quantum-interference-device biosusceptometry, Magnetic resonance imaging

## Abstract

Although the biomarker carcinoembryonic antigen (CEA) is expressed in colorectal tumors, the utility of an anti-CEA-functionalized image medium is powerful for *in vivo* positioning of colorectal tumors. With a risk of superparamagnetic iron oxide nanoparticles (SPIONPs) that is lower for animals than other material carriers, anti-CEA-functionalized SPIONPs were synthesized in this study for labeling colorectal tumors by conducting different preoperatively and intraoperatively *in vivo* examinations. In magnetic resonance imaging (MRI), the image variation of colorectal tumors reached the maximum at approximately 24 h. However, because MRI requires a nonmetal environment, it was limited to preoperative imaging. With the potentiality of *in vivo* screening and intraoperative positioning during surgery, the scanning superconducting-quantum-interference-device biosusceptometry (SSB) was adopted, showing the favorable agreement of time-varied intensity with MRI. Furthermore, biological methodologies of different tissue staining methods and inductively coupled plasma (ICP) yielded consistent results, proving that the obtained *in vivo* results occurred because of targeted anti-CEA SPIONPs. This indicates that developed anti-CEA SPIONPs owe the utilities as an image medium of these *in vivo* methodologies.

## Background

Colorectal tumors, which are caused by uncontrolled cell growth in the colon or rectum [1], have constituted the third most commonly diagnosed cancer in the world, especially in developed countries [2]. In screening methods, a stool occult blood test is usually performed when the patient has experienced symptoms such as unusual bowel habits. When the result is positive, flexible sigmoidoscopy, barium enema, or colonoscopy is further applied. Because of discomfort and risks, such as the colonic perforation that can occur in these invasive methods, noninvasive methods [3], such as computed tomography (CT), positron emission tomography (PET), and magnetic resonance imaging (MRI), are alternatively used to image not only the primary colorectal tumor but also metastatic tumors in other organs.

Two approaches can enhance the sensitivity and specificity of these medical imaging procedures [3]. The first approach is the multimodality of structural imaging and functional imaging, such as the CT/PET and MRI/PET. The second is based on image contrast media using bioprobes. Here, the image contrast media are the radioactive materials for CT and PET and the superparamagnetic materials for MRI. It is well known that these radioactive media and methodologies entail a biological risk and that the clinically popular gadolinium medium of MRI superparamagnetic materials induces the side effect of kidney disease [4]. Because iron oxide materials have a low risk of toxicity [5], superparamagnetic iron oxide nanoparticles (SPIONPs) coated with bioprobes have been developed for highly specific labeling [6] of targeted tumors in examining [7] and treating [8] tumors.

Because carcinoembryonic antigen (CEA) is expressed in colorectal cancer [9], it is a useful indicator for treatment progress according to the decreasing CEA level in plasma [10]. Therefore, anti-CEA SPIONPs were developed as the contrast medium of MRI for colorectal cancer. However, because MRI requires a no-metal and shielded environment, as well as the patient to lie inside a coil, the procedure is limited to preoperative examination rather than intraoperative examination. Therefore, the multimodality image contrast media of preoperative and intraoperative examinations have emerged recently, for example, nanoparticles used in preoperative MRI with an additional near-infrared fluorescent [11] indicator for intraoperative optical imaging or an additional radioactive indicator for an intraoperative gamma imaging [12]. However, synthesizing various functional indicators on nanoparticles increases not only the cost but also the toxicity risk.

To accommodate the needs of preoperative and intraoperative examinations using simple SPIONPs without additional indicators, the superior magnetic characteristics of SPIONPs should be examined for conducting different *in vivo* examinations. For example, the paramagnetic or superparamagnetic characteristics of SPIONPs have been used for performing the image contrast of MRI [13]. Similarly, the nonlinear response of SPIONPs was developed to reveal SPIONP distributions by magnetic particle imaging (MPI). However, the field of view of MPI currently is quite small, for example, the beating heart of a mouse [14,15]. Recently, a scanning superconducting-quantum-interference-device biosusceptometry (SSB) system, possessing the advantage of an ultrasonic-like operation, was developed to track SPIONPs noninvasively without using bioprobes in animals [16,17]. The mechanism entails examining the in-phase component of the AC susceptibility of SPIONPs.

In this work, to validate the simple anti-CEA-functionalized SPIONPs demonstrating the ability to label colorectal tumors, anti-CEA-functionalized SPIONPs were synthesized and injected into mice implanted with colorectal tumors for MRI and SSB examinations *in vivo*.

## Methods

The Animal Care and Use Committee of National Taiwan University approved all experimental protocols (No. 20110009), named 'Development of Core-technologies and Applications of Nano-targeting Low-field Magnetic Resonance Imaging.’ All experiments were conducted according to the animal care guidelines of the university.

**Figure 1:**
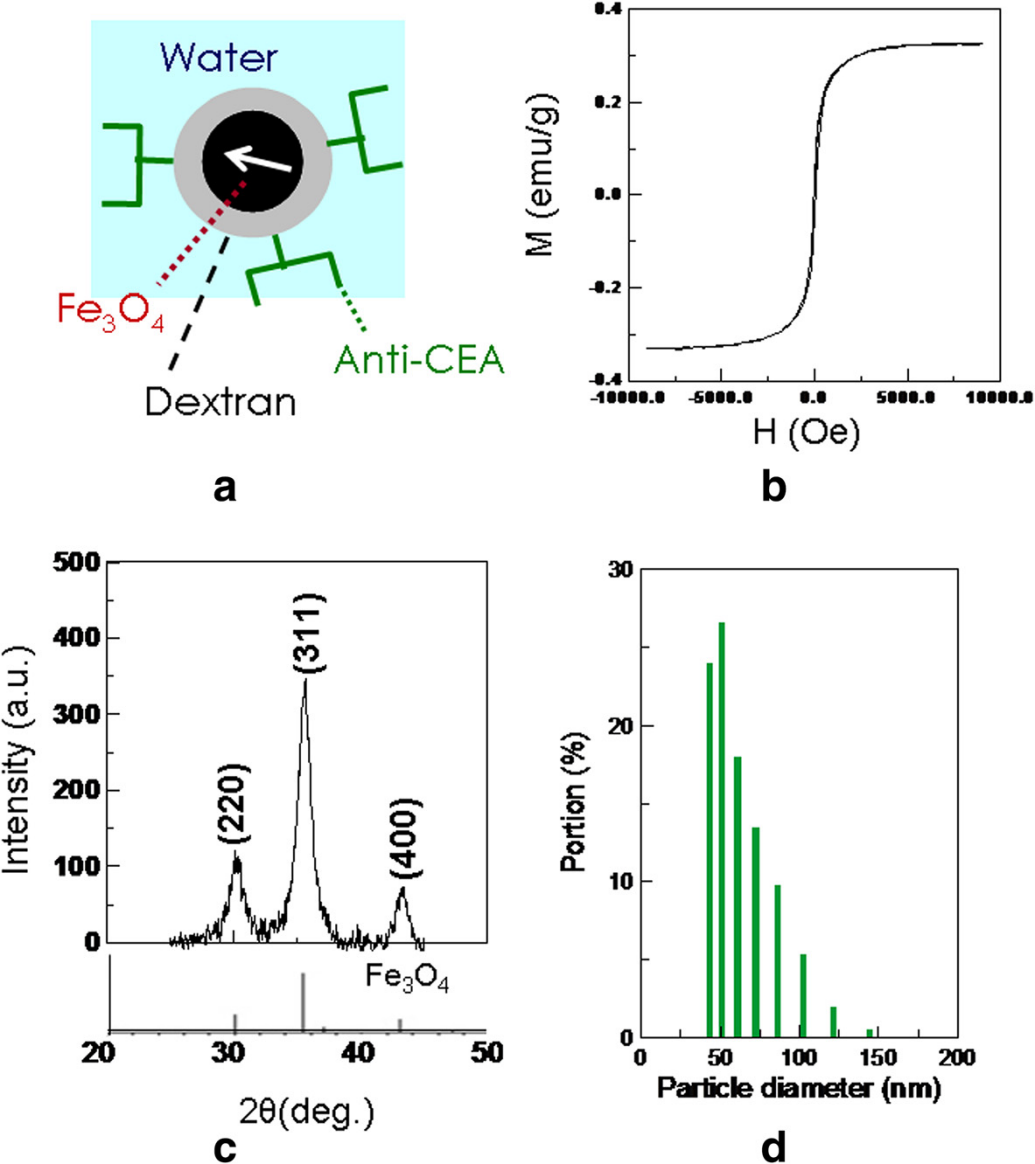
**Characterization of anti-CEA MFs. (a)** The structural scheme of anti-CEA MFs. **(b)** Magnetization properties of anti-CEA MFs. **(c)** The PXRD pattern of the crystalline SPIONPs. **(d)** Distribution of the hydrodynamic diameter of SPIONPs.

The used magnetic fluids (MFs), as shown in Figure 1a, were composed of anti-CEA SPIONPs and water solvents. Anti-CEA SPIONPs were synthesized from Fe_3_O_4_ SPIONPs without any antibody coating (MagQu Corp., Taipei, Taiwan). By oxidizing the dextran coating of Fe_3_O_4_ SPIONPs with NaIO_4_ to create aldehyde groups (-CHO) [18], the dextran reacted with the anti-CEA antibodies (10C-CR2014M5, Fitzgerald, Acton, MA, USA) through -CH = N- to conjugate the anti-CEA antibodies covalently. Performing magnetic separation then separated the unbound antibodies from the MFs. The used MFs were characterized according to magnetic characteristics using a vibration sample magnetometer (Model 4500, EG&G Corp., San Francisco, CA, USA), according to particle size by dynamic laser scattering (Nanotrac 150, Microtrac Corp., Montgomeryville, PA, USA), and according to magnetic composition using a diffractometer (D-500, Siemens Corp., Munich, Germany) for powder X-ray diffraction.

For implanting the colorectal tumors, the injections of the CT-26 cell line were processed through the skin on the backs of 8-week-old mice. Three weeks later, 0.06 emu/g and 100 μl of anti-CEA SPIONPs in water were injected into the tail veins of five mice. Two mice, mouse 1 and mouse 2, were examined using SSB and MRI magnetic instruments. The SSB examination schedule was at the 0th, 14th, 26th, 40th, 68th, and 92nd hours for mouse 1 and at the 0th, 8th, 20th, and 42nd hours for mouse 2. The MRI examination schedule was 4 h later than each SSB examination time. Here, 0th represents the time before injection. Proving that the anti-CEA SPIONPs were bound to the tumor tissue required determining the Fe amount using inductively coupled plasma (ICP) and well-known tissue staining methods, such as hematoxylin and eosin (HE) staining, Prussian blue (PB) staining, anti-CEA staining, and cluster of differentiation 31 (CD 31) staining, to examine the tumor tissue of three mice, mouse 3, mouse 4, and mouse 5, which were euthanized at the 0th, 24th, and 98th hours, respectively.

**Figure 2:**
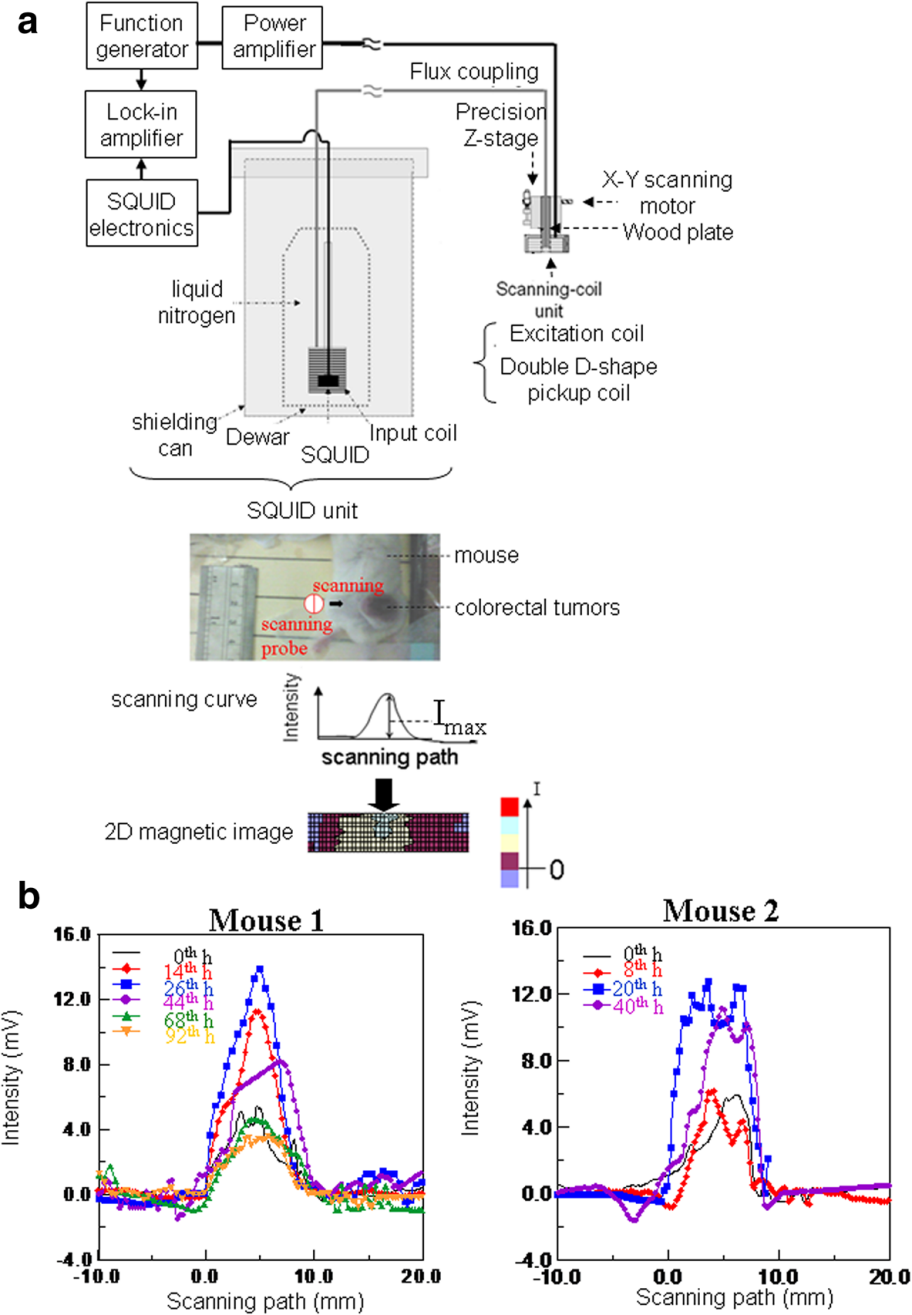
**SSB examination. (a)** The schemes of SSB and its examination of a mouse with a colorectal tumor on its back. **(b)** The scanning curves at maximum intensity.

The SSB scheme, a novel magnetic handy probe as shown in Figure 2a, has two major parts, the superconducting quantum interference device (SQUID) unit and the scanning probe unit. The SQUID unit was composed of a high-*T*_c_ SQUID sensor (JSQ GmbH, Jülich, Germany) surrounded by an input coil, cooled in liquid nitrogen, and shielded in a set of shielding cans. The scanning probe unit was composed of excitation and pickup coils, which were moved by a three-axial step motor. The shielded copper wires were connected to the pickup coils of the scanning probe unit and the input coil of the SQUID unit for flux transfer. Therefore, SSB has the superior advantages of convenient magnetism measurement by moving the scanning probe along any sample contour. Besides, the measured signal intensity could be amplified by a suitable transfer design. Both are opposite to the complex alignment of the sample under a small SQUID sensor and have a sensitivity limited by the mechanism of the cooing Dewar and the shielding can for a general SQUID system. In addition to the superior sensitivity of several picotesla, the excitation field of 400 Hz and 120 Oe was determined to be safe for animals because of their frequency-strength product being smaller than the criteria of 4.85 × 10^8^ kA/m s [19]. Under the alternating-current (AC) magnetic excitation field, the AC susceptibility of samples resulted in the AC magnetism for SSB examination.

During the measurement process, the test mice were initially anesthetized, and their back tumors were then covered by a plastic plate with a tumor-fit hole to fix the relative orientation and distance of the tumor from the scanning probe unit. The scanning probe unit scanned the entire tumors in several scanning paths with a vertical interval of 0.1 mm. Thus, a magnetic image for the tumor could be constructed, as shown in Figure 2a. SPIONPs under AC field excitation generally expressed the characteristics of AC susceptibility. Therefore, the SSB signal from the in-phase component of the AC susceptibility of SPIONPs was in proportion to the SPIONP concentration [16].

The 3-T MRI (Bruker Biospec System, Karlsruhe, Germany) and a volume coil were used for T2-weighted images. In parallel with the arrangement of the anesthetized mouse, a long tube filled with deionized (DI) water was inserted as the intensity reference to dismiss the instrument drift at various times. Producing the coronal images of each entire mice body at 2-mm intervals required nearly 2 h.

In general, the uniformity of the static field and gradient field is distorted by SPIONPs, resulting in the dephasing of the proton nuclear spin and, subsequently, the reduction of nuclear magnetic resonance (NMR) intensity induced by the pulse field of MRI [20]. Hence, the labeled tumor cells using bound SPIONPs expressed a darker image. Therefore, SPIONPs were the contrast agent of the MR images.

For ICP examination (EVISA Instruments, PE-SCIEX ELAN 6100 DRC, High Valued Instrument Center, National Science Council, Kaohsiung, Taiwan), two pieces of tumor tissue from one euthanized mouse were both weighted by a 0.1-g weight and then dissolved entirely in a HNO_3_ solution at a concentration of 65%; they were then diluted and examined. To evaluate the incorporation of an anti-CEA SPIONP quantity into the tumor tissue, the difference of Fe concentration between the varied post-injection and pre-injection times at the 0th hour was expressed as Δ*C*_Fe_ (ppm).

The tissue staining was processed (Laboratory Animal Center, National Taiwan University, Taipei, Taiwan), and the × 400 magnification of the optical images was observed using a light microscope. HE staining, PB staining, anti-CEA staining, and CD 31 staining were performed to identify the tumor tissue, Fe element distribution, and anti-CEA SPIONP distribution; and the degree of tumor angiogenesis was related to the transportation of anti-CEA SPIONPs.

## Results and discussion

Figure 1b shows the curve of the magnetism-applied field (*M*-*H*) curve of anti-CEA SPIONPs. Based on the ultralow hysteresis in the *M*-*H* curve, the anti-CEA SPIONPs expressed superparamagnetic characteristics. Furthermore, the X-ray pattern of the anti-CEA SPIONPs in Figure 1c depicts the crystal structure of anti-CEA SPIONPs obtained by X-ray diffraction. The major peaks correspond with the standard X-ray pattern of Fe_3_O_4_ (JCPDS card no. 65–3107), verifying that the magnetic material was Fe_3_O_4_, a magnetic iron oxide (IO). In addition, the distribution of the hydrodynamic diameter, as shown in Figure 1d, indicates that anti-CEA SPIONPs have a mean diameter of 50 ± 2 nm. In other words, anti-CEA SPIONPs belong to the so-called 'ultrasmall superparamagnetic iron oxides (USPIOs)’ [21].

**Figure 3:**
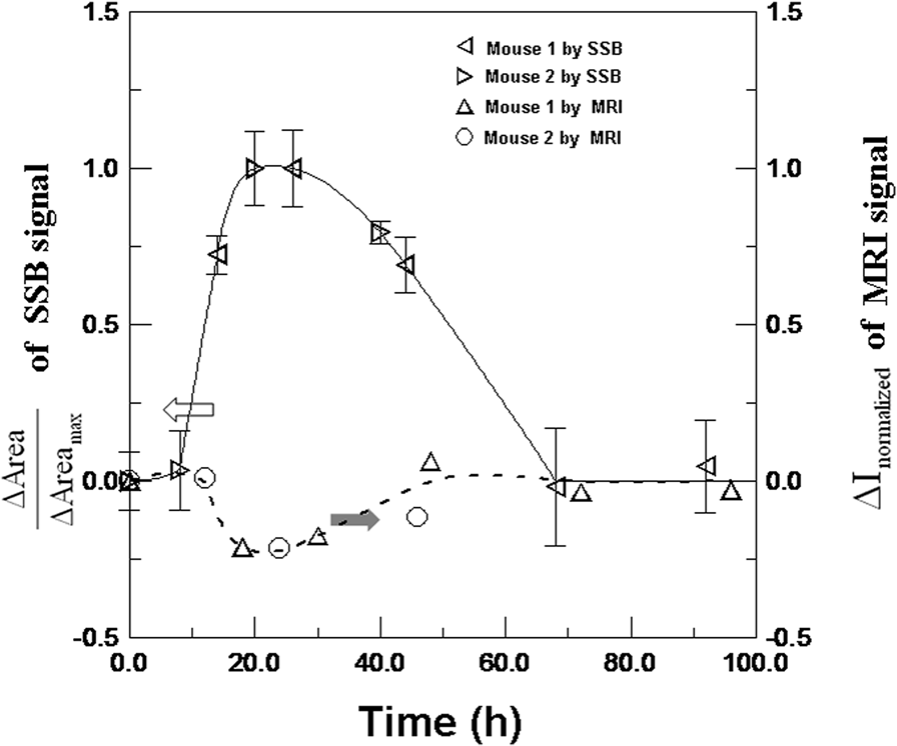
**Comparison between** Δ**Area/Area**_**max **_**by SSB and *I***_**normalized **_**by MRI for mouse 1 and mouse 2.**

An entire colorectal tumor implanted in an anesthetized mouse was scanned using the SSB scanning probe for 4 min. After each scanning, a scanning curve was obtained, as shown in the inset of Figure 2a. Among all scanning curves at a time point, the scanning curve with the largest *I*_peak_, the maximum intensity, was selected as a representative for comparison with other *I*_peak_ at various times, as shown in Figure 2b. In Figure 2b, both *I*_peak_ and the peak width of the scanning curve increased from the 0th hour, achieved the maximum at the 26th hour for mouse 1 and the 20th hour for mouse 2, and decreased to levels similar to those at the 0th hour. Therefore, the reliable area of the scanning path, 'Area,’ was used to analyze the magnetism of the entire tumors by adding the products of the scanning step and the intensities that were larger than the half of *I*_peak_. Here, the intensities that were smaller than the half of *I*_peak_ were skipped because of the significant repeatability errors occurring under particular experimental conditions such as the arrangement of the mouse and mouse breath. Consequently, the maximum Area of mouse 1 and mouse 2 occurred separately at the 26th hour and the 20th hour. To prove the reliability of the SSB results by comparing them with the MRI results, the normalized parameter ΔArea/Area_max_ was used to express the magnetic enhancement using anti-CEA SPIONPs on a colorectal tumor, as shown in Figure 3. The examination of magnetic labeling of tumors by SSB, as shown in Figure 3, indicated that the accumulation of anti-CEA SPIONPs reached the highest level and gradually dissipated to the initial level at approximately the 72nd hour. Because anti-CEA SPIONPs showed not only the in-phase component of the AC susceptibility for SSB examination but also the superparamagnetic properties for MRI contrast imaging, hence, the dynamic amount variation of anti-CEA SPIONPs binding to colorectal tumors could be verified by the *I*_normalized_ variation of the MR image with time.

**Figure 4:**
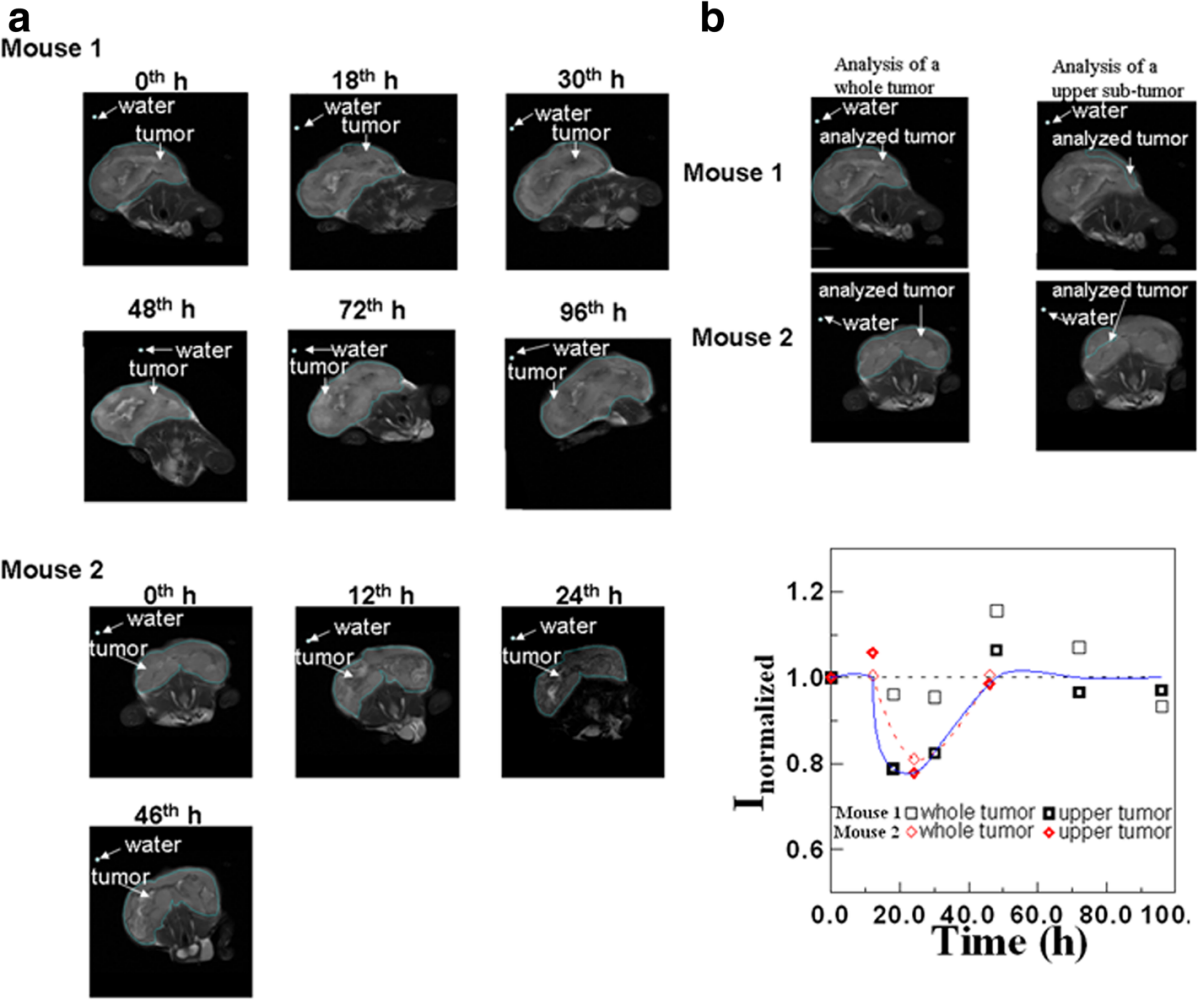
**MRI examination. (a)** MR images of mouse 1 and mouse 2 at various examination times. **(b)** The analytical comparison between the image intensities of the entire and upper tumor regions. The figure inset shows the time variations of different image intensities of mouse 1 and mouse 2, analyzed in the entire and upper tumor regions.

Figure 4a shows the representative MR images for the colorectal tumors of mouse 1 and mouse 2 at various times. Here, the entire tumor was marked with a blue outline and selected for analysis, and the DI water in the tube was also used for comparison. Based on observation, the tumor of mouse 2 became significantly dark at the 24th hour and then recovered to brightness at the 0th hour. In addition, the normalized intensity, *I*_normalized_, was defined as the ratio of the average intensity of the selected region over that of DI water. The variation of *I*_normalized_ for the entire tumor was analyzed, as shown in Figure 4b, indicating that *I*_normalized_ for the entire tumor around the first day reached the minimum for mouse 2. However, for mouse 1 at the 18th and 30th hours, close to the first day, *I*_normalized_ for the entire tumor had no significant difference from at the 0th hour. These MRI results varied slightly from those of the SSB examination. Therefore, the analyzed tumor in the MR images was chosen as the upper region instead of the entire tumor, as depicted in Figure 4b. Consequently, the variation of *I*_normalized_ for both mouse 1 and mouse 2 generally reached the minimum at approximately the 24th hour. Furthermore, Δ*I*_normalized_ of the local upper region, defined as the difference of *I*_normalized_ between post-injection and the 0th hour, was used to evaluate the image brightness variation of the parts of the tumors that occurred because of the accumulation of anti-CEA SPIONPs, as depicted in Figure 4b. In comparison with ΔArea/Area_max_ by SSB, Figure 3 shows that the magnetic labeling of colorectal tumors using anti-CEA SPIONPs could be examined by both SSB and MRI because of the same variation trend of ΔArea/Area_max_ by SSB and Δ*I*_normalized_ by MRI at various times. The varied signs of plus and negative properties were due to the distinct magnetic characteristics of anti-CEA SPIONPs and the enhancement of AC magnetic susceptibility [16] for SSB different from the distortion of DC imaging field [20] for MRI. In addition, regarding tumors implanted in the mouse flank in other works, the similarity of this time-varied trend [22] demonstrated the reasonability of using specific probe-mediated SPIONPs in labeling tumors.

Furthermore, regarding the mentioned favorable agreement between the SSB results and the MRI results of the upper region of a labeled tumor rather than the entire region, it was explained as follows. In tumor development, most of the scab tumors were possibly fiber tissue or dead tumor cells in the tumor center; however, the upper region, in which more distribution of live tumor cells occurred around the tumor center [23], constituted live cells for binding anti-CEA coating SPIONPs. Hence, for colorectal tumors labeled with developed anti-CEA SPIONPs, a two-dimensional (2D) magnetic image (Figure 2a) of SSB was in charge of *in vivo* screening initially and intraoperative positioning finally, and MRI worked for only preoperative imaging.

**Figure 5:**
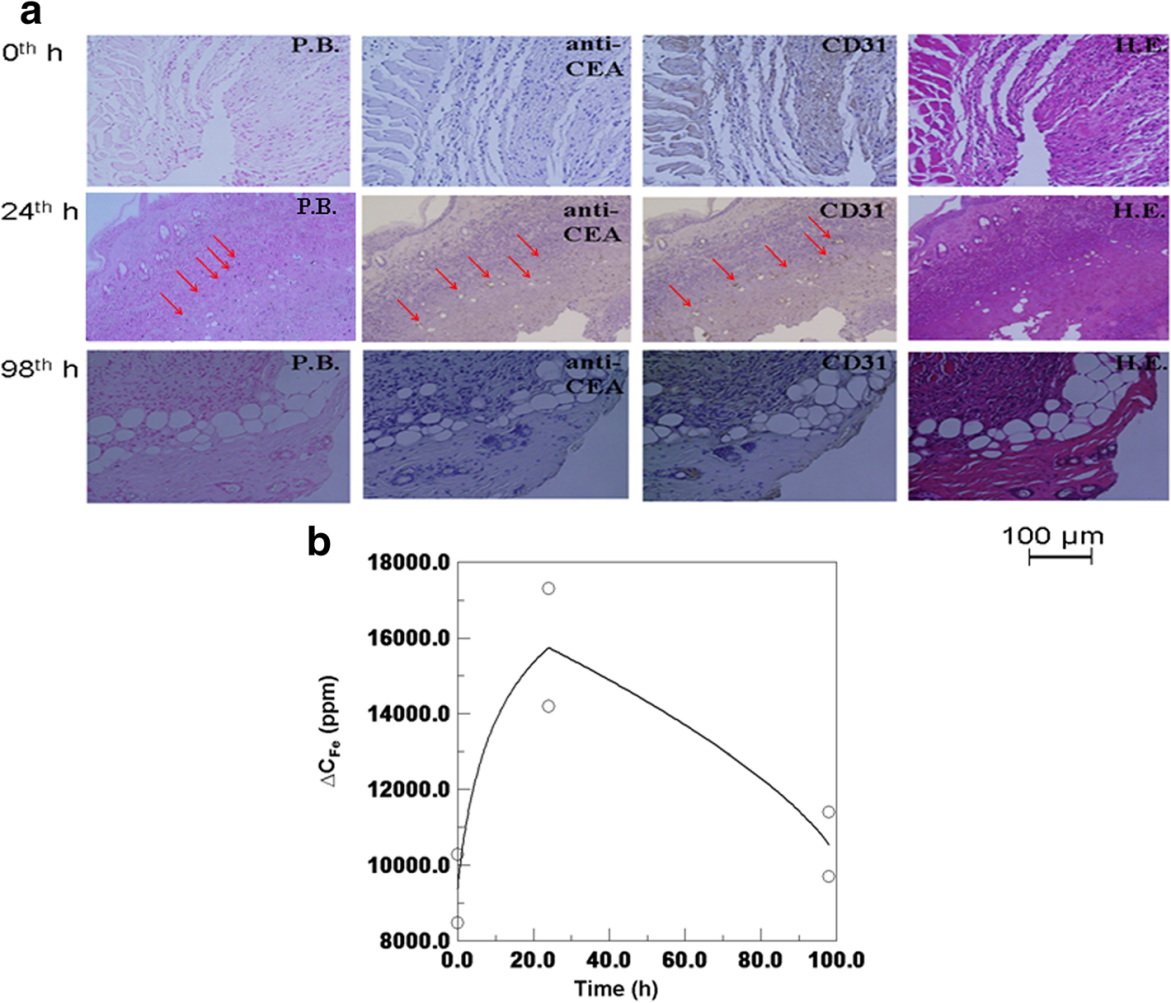
**Biological results of the tumors of mouse 3, mouse 4, and mouse 5. (a)** Tissue staining methods of HE staining, PB staining, anti-CEA staining, and CD 31 staining. **(b)** Iron amount by ICP. The circles are data points obtained from the measured results of two tissues.

Furthermore, these magnetic characteristics of a tumor labeled with anti-CEA SPIONPs were verified using the gold standard of biological assays, tumor tissue staining, and ICP. Figure 5a shows the times at which the colorectal tumors were removed from mouse 3 (at the 0th hour), mouse 4 (at the 24th hour), and mouse 5 (at the 98th hour), by PB staining, immunostaining of anti-CEA protein, CD 31 staining, and HE staining. As shown in the photo of HE staining, these cells also developed in proximity to disorganized architectures because of the increased ratio of nuclei to cytoplasm. This indicated that these tissues were obtained from tumors. Furthermore, there are significant blue spots (arrows), representative of iron elements, in the PB photo and brown spots (arrows) in the anti-CEA and CD 31 photos at the 24th hour, but not at the 0th and 98th hours. In addition, the distribution consistency of the blue spots in the PB photos, as well as the brown spots in both the anti-CEA and CD31 photos, indicated that the tumors were labeled by these anti-CEA SPIONPs rather than by biodegraded iron ions through the transportation of microvessels. This also confirmed that selecting the upper tumor region was more suitable than selecting the entire tumor for MRI because of the live zone of the tumor with both microvessels and anti-CEA SPIONPs.

Figure 5b shows the variation of the average iron amounts in tumor tissues reaching the highest level at the 24th hour and recovering at the 98th hour to the initial level at the 0th hour. Therefore, the various amounts of both anti-CEA SPIONPs by tissue staining and Fe element distribution by ICP correspond with the magnetic results obtained by SSB and MRI.

## Conclusions

In summary, anti-CEA SPIONPs with simple structures demonstrated superior magnetic characteristics for examining colorectal tumors *in vivo*. Because the dynamics of magnetic labeling was consistent with biological phenomena by tissue staining and ICP, the feasibility of examining targeted colorectal tumors by SSB and MRI was proved. This indicates that this type of anti-CEA SPIONP can be used in a complete series of medical applications, such as *in vivo* screening and intraoperative positioning, by SSB and conducting preoperative examination by MRI.

## Competing interests

The authors declare that they have no competing interests.

## Authors’ contributions

JJC designed and performed the SSB experiments and wrote the manuscript. KWH prepared the animal experiments and proposed the protocol of animal test. ITL contributed to MR imaging. HEH, HCY, and CYH participated in the design of the study and discussion. All authors read and approved the final manuscript.
